# Identification of symplasmic domains in the embryo and seed of *Sedum acre* L. (Crassulaceae)

**DOI:** 10.1007/s00425-016-2619-y

**Published:** 2016-11-25

**Authors:** Justyna Wróbel-Marek, Ewa Kurczyńska, Bartosz J. Płachno, Małgorzata Kozieradzka-Kiszkurno

**Affiliations:** 1 Department of Cell Biology, University of Silesia, Jagiellońska 28, 40-032 Katowice, Poland; 2 Department of Plant Cytology and Embryology, Jagiellonian University in Kraków, Gronostajowa 9, 30-387 Kraków, Poland; 3 Department of Plant Cytology and Embryology, University of Gdańsk, Wita Stwosza 59, 80-308 Gdańsk, Poland

**Keywords:** Embryogenesis, Plasmodesmata, Symplasmic communication, Symplasmic domains

## Abstract

**Our study demonstrated that symplasmic communication between** ***Sedum acre*** **seed compartments and the embryo proper is not uniform.**

The presence of plasmodesmata (PD) constitutes the structural basis for information exchange between cells, and symplasmic communication is involved in the regulation of cell differentiation and plant development. Most recent studies concerning an analysis of symplasmic communication between seed compartments and the embryo have been predominantly performed on *Arabidopsis thaliana*. The results presented in this paper describe the analysis of symplasmic communication on the example of *Sedum acre* seeds, because the ultrastructure of the seed compartments and the embryo proper, including the PD, have already been described, and this species represents an embryonic type of development different to *Arabidopsis*. Moreover, in this species, an unusual electron-dense dome associated with plasmodesmata on the border between the basal cell/chalazal suspensor cells and the basal cell/the endosperm has been described. This prompted the question as to whether these plasmodesmata are functional. Thus, the aim of this study was to describe the movement of symplasmic transport fluorochromes between different *Sedum* seed compartments, with particular emphasis on the movement between the basal cell and the embryo proper and endosperm, to answer the following questions: (1) are seeds divided into symplasmic domains; (2) if so, are they stable or do they change with the development? The results have shown that symplasmic tracers movement: (a) from the external integument to internal integument is restricted; (b) from the basal cell to the other part of the embryo proper and from the basal cell to the endosperm is also restricted; (c) the embryo is a single symplasmic domain with respect to molecules of a molecular weight below 0.5 kDa.

**Electronic supplementary material:**

The online version of this article (doi:10.1007/s00425-016-2619-y) contains supplementary material, which is available to authorized users.

## Introduction

The cytoplasm of individual plant cells is connected by PD, which are dynamic structures capable of selectively controlling the movement of different molecules between the cells (Burch-Smith et al. 2011). This movement of molecules through PD is called symplasmic communication. Changes in the number and structure of PD, modifications of individual PD ultrastructure and their capacity in regulating the movement of substances between the cells during plant growth and development influence the symplasmic communication within the plant body. Thus, the degree of symplasmic communication is different in various organs/tissues and depends on the state of development, conditions of the growth and environmental factors (Benitez-Alfonso 2014; Kragler 2013).

Changes in symplasmic communications within the plant body appear from the earliest stages of plant development. Studies on *Torenia fournieri* have shown that even the reproductive cells are isolated symplasmically and maturation of the embryo sac is correlated with the gradual decline in symplasmic continuity between the egg cell and the central cell (Han et al. 2000). During embryogenesis, the differences in symplasmic communication correlate with organ development and tissue differentiation, as has been shown for *Arabidopsis thaliana* zygotic embryos and androgenic embryos of *Hordeum vulgare* (Kim et al. 2002; Wrobel et al. 2011).

Studies on the mechanisms controlling embryogenesis are of primary importance, as this process is the most crucial one from the developmental point of view (Goldberg et al. 1994; Souter and Lindsey 2000). With regards to seeds, and their nutrition during the development and exchange of developmental signals, symplast and apoplast are involved in the substance exchange (van Dongen et al. 2003; Zažímalová et al. 2010; Yeung and Meinke 1993; Kawashima and Goldberg 2010; Ruan et al. 1997; Patrick and Offler 2001; van Dongen et al. 2003; Stadler et al. 2005; Liu et al. 2015).

Most recent studies concerning physiology and transport, including symplasmic communication between different seed compartments and within the embryo proper, have mainly been performed on *Arabidopsis* (Kim and Zambryski 2005; Kim et al. 2005; Babu et al. 2013; Liu et al. 2015), and only a few papers have focused on other flower plants (Lee and Yeung 2010; Zhao et al. 2013). Therefore, more studies on other species are needed. This would enable some generalisations concerning the role of symplasmic communication during embryogenesis. This is especially important because *A. thaliana* represents only one type of embryonic development where embryo differentiation follows the classical *Capsella* variation of the Onagrad type (Mansfield and Briarty 1991).

The studies presented in this paper concern an analysis of symplasmic tracers movement between different seed compartments on the example of *Sedum acre* (family Crassulaceae), which represents a type of embryonic development different to *Arabidopsis* (Kozieradzka-Kiszkurno and Bohdanowicz 2006; Kozieradzka-Kiszkurno et al. 2011b). The representatives of the family Crassulaceae undergo the Caryophyllad type of embryonic development, and in case of many taxa from this family, the haustorial suspensor consists of a giant basal cell with a micropylar haustorium and a few smaller chalazal cells (Kozieradzka-Kiszkurno and Bohdanowicz 2003; Kozieradzka-Kiszkurno et al. 2011b, 2012). Moreover, the ultrastructure of *S. acre* embryo and seed is already well described, including the PD between different seed compartments (Kozieradzka-Kiszkurno and Bohdanowicz 2010; Kozieradzka-Kiszkurno et al. 2011a). Also, these PD are wider than typical PD in angiosperms and are covered by a dome of an electron-dense material (Kozieradzka-Kiszkurno and Bohdanowicz 2010; Kozieradzka-Kiszkurno et al. 2011a). In contrast to *Arabidopsis*, PD between the endosperm and suspensor have been recorded in *Sedum* (Kozieradzka-Kiszkurno and Płachno 2012). The question as to whether they are functional and whether symplasmic transport exists remains unanswered.

Thus, the aim of the present study was to trace the movement of symplasmic transport fluorochromes, which should reflect the symplasmic communication between different seed compartments and embryos on the example of *S. acre* to answer the following questions: (1) Is there any symplasmic communication between seed compartments? (2) What does symplasmic communication look like on the borders: the basal cell/the embryo proper and between the basal cell and the endosperm?

## Materials and methods

### Plant material

Developing *S. acre* L. seeds were collected during two successive growing seasons from plants grown in natural habitats around Katowice in southern Poland.

For microscopic analyses, the seeds were isolated from ovaries in water using preparation needles. In the present study, about 200 seeds were treated with fluorochromes. The procedure for the seed histology (semi-thin sections) was the same as used earlier by Kozieradzka-Kiszkurno and Bohdanowicz (2006).

### Fluorochromes

Symplasmic communication was examined using the following fluorochromes: CMNB-caged fluorescein [fluorescein bis-(5-carboxymethoxy-2-nitrobenzyl) ether, dipotassium salt], HPTS (8-hydroxypyrene-1,3,6-trisulfonic acid, trisodium salt), HPTSA (8-acetoxypyrene-1,3,6-trisulfonic acid, trisodium salt) and LYCH (Lucifer Yellow CH). The solutions of HPTS, and HPTSA (see also Wrobel et al. 2011) were prepared by dissolving 5 mg of the fluorochrome in 1 ml of demineralised water. LYCH solution was made by dissolving 2.5 mg of the fluorochrome in 1 ml of demineralised water. To prepare the solution of CMNB-caged fluorescein (CMNB-F), a stock solution was first made (fluorochrome was dissolved in 1 ml of 0.2% dimethyl sulfoxide; procedure according to Martens et al. 2004). Then, the stock solution was dissolved in demineralised water to a final concentration of 0.01%. HPTS and LYCH are not membrane-permeable; thus, the plant material had to be injured to introduce these fluorochromes into the cell cytoplasm. CMNB-caged fluorescein and HPTSA can move through the cell membrane; hence, their application was not invasive. However, both fluorochromes in their membrane-permeable forms are not fluorescent. CMNB-F must be uncaged, as described below, to release the form that is fluorescent but not membrane-permeable (fluorescein). The distribution of that form is monitored further. HPTSA is an acetic derivative of HPTS. After entering the cytoplasm, acetic groups of HPTSA are cleaved by intercellular, non-specific esterases, and the fluorescent, but not membrane-permeable form of the fluorochrome (HPTS) is released and monitored further.

### Analysis of symplasmic tracer distribution

#### *CMNB*-*caged fluorescein* (Thermo Fisher Scientific, F-7103, after uncaging MW = 332 Da)

Studied seed/embryo compartments (depending on study needs) were incubated for 60–90 min (time of incubation was determined experimentally and was similar to published data: Wrobel et al. 2011; Kulinska-Lukaszek and Kurczynska 2012) in an excess of the fluorochrome and rinsed in demineralised water (to remove unincorporated dye). Then, they were placed upon a microscopic slide (in demineralised water) and covered with a cover glass. Uncaging of CMNB-F was performed using either an epifluorescence microscope (Nikon Eclipse Ni) or a confocal laser scanning microscope (CLSM, Olympus). The selected part of the studied seed compartments was illuminated with either UV + Vis lamp (epifluorescence microscope) using a UV-2A filter (excitation wavelength 330–380 nm) for 30 s or an LD laser (CLSM) emitting 405-nm wavelength for 60–120 s. Time of activation was determined experimentally (the shortest activation time, after which the illuminated area gave a positive signal that was observed; for CLSM the power of LD laser emitting 405 nm wavelength for photoactivation was 60%, and for imaging, the-power of argon laser emitting 488-nm wavelength was set to 8%). Such a procedure released fluorescein within the studied cells. The region of illumination independently of the microscope type (epifluorescent and CLSM) was selected under the bright-field illumination and it was also checked in the fluorescence channel of 520 nm for epifluorescence microscope and 500–600 nm for CLSM. The area for illumination was selected either by closing a field aperture of epifluorescence microscope or marking the region of interest (ROI) in CLSM software (FluoView, Olympus). Uncaging of fluorescein was performed in different regions of studied compartments and at different stages of their development. The spatial pattern of uncaged fluorescein distribution was monitored and photographed immediately after uncaging and at 5-min intervals during the following hour. It must be noted that, some steps in the procedure with CMNB-caged fluorescein were done according to Goodwin and Cantrill (1999), Martens et al. (2004) and Liesche and Schulz (2015).

#### *HPTS* (Thermo Fisher Scientific, H-348, MW = 520 Da)

**Fig. 1:**
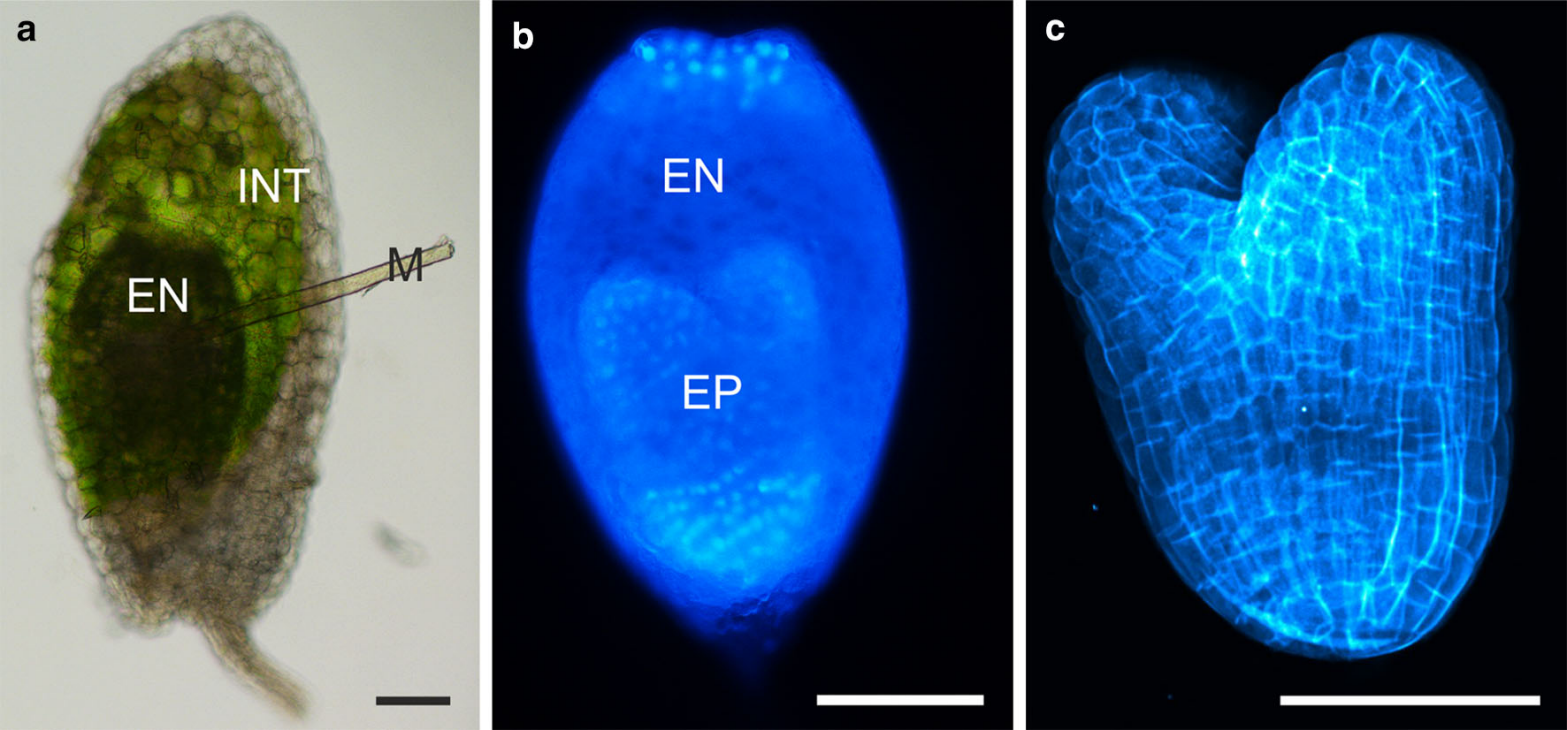
Imaging of microcapillary introducing and control stainings. Fluorochromes which are not membrane-permeable were introduced to seed cells by a microcapillary. **a** In our study, two control stainings were performed: DAPI (**b**) and calcofluor white (**c**). Both fluorochromes penetrated the plant material within 15 min and proved that there were no physical obstacles that could prevent the movement of symplasmic transport fluorochromes within seeds. *EN* endosperm, *EP* embryo proper, *INT* integument (external and internal), *M* microcapillary. Image “**a**” was taken under a bright-field microscope, image “**b**” was taken under an epifluorescence microscope and image “**c**” is a CLSM *z*-stack projection consisting of at least five optical sections. *Scale bar* 100 μm

Whole seeds and the isolated endosperm (enclosing the embryo with suspensor) were pre-treated with DDG and placed in an excess of the fluorochrome and were injured with a microcapillary with an outer diameter ranging between 3 and 7 µm (Fig. 1a). Then, the microcapillary was removed and the plant material was incubated in the fluorochrome for 30–90 min. Thereafter, the plant material was rinsed in demineralised water (to remove unincorporated dye) and placed upon a microscopic slide and covered with a cover glass. The distribution of HPTS was analysed using an epifluorescence microscope (Nikon Eclipse Ni). The fluorochrome was excited by a B-2A filter (excitation wavelength 450–490 nm). Under such conditions, the emission of HPTS was collected at 520 nm, producing a green–yellow fluorescence.

#### *HPTSA* (Carbosynth, EA 45175, after esterase cleavage MW = 520 Da)

To examine symplasmic communication, the endosperm (enclosing the embryo with suspensor) was isolated from the seed and immersed in an excess of HPTSA solution for 30–90 min. After that, the plant material was rinsed in demineralised water (to remove unincorporated dye) and examined under an epifluorescence microscope or CLSM. Apart from that, inflorescence stems were also immersed in the fluorochrome for varying periods of time (between 80 min and 48 h). Then, the seeds were isolated from the ovaries, rinsed in demineralised water and examined under CLSM. Detection of the fluorochrome was identical to the conditions used in the case of HPTS (epifluorescence microscope; excitation wavelength: 450–490 nm, emission wavelength: 520 nm) or fluorescein (CLSM; excitation wavelength 488 nm, emission wavelength 500–600 nm).

#### *LYCH* (Thermo Fisher Scientific, L1177, MW = 443 Da)

Lucifer Yellow CH solution was introduced into DDG-treated seeds or embryos with a microcapillary (see “HPTS”). Observation of LYCH distribution was carried out under conditions which were identical to those used for HPTS in case of epifluorescence microscope. Using CLSM, Lucifer yellow was excited by 458-nm wavelength and its emission was collected at 530–600 nm wavelength giving yellow fluorescence.

### Acquisition of images

In case of CLSM procedure for receiving images consisted of determination of the upper and lower *z*-limit for image stacks acquisition. To prevent the collection of autofluorescence, the CLSM parameters were adjusted so that a photomultiplier collected only fluorescence of used fluorochromes. Thus, the power of argon laser (for 458 and 488 nm wavelength) and photomultiplier voltage were set to the lowest position in which fluorescence of fluorochromes was still visible but autofluorescence was excluded.

### Autofluorescence

The autofluorescence of seeds and embryos at all developmental stages was checked and because there were not quantitative and qualitative differences between them, the representative photos are presented in Table 1. Well visible was the autofluorescence of chlorophyll (red) in the integument. Almost invisible was the autofluorescence of embryos (white lines indicate their shapes).

**Table 1:**
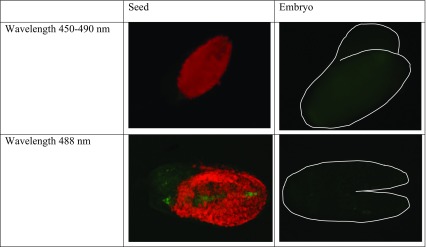
Autofluorescence of *Sedum acre* seeds and embryos observed at different wavelengths used for fluorochrome distribution analysis

### DDG

Due to the fact that injuries might lead to callose deposition at plasmodesmata, plant material treated with HPTS and LYCH was pre-treated with 0.1 mM solution of DDG (2-deoxy-d-glucose; Sigma-Aldrich, D8375) in demineralised water, for 30–90 min (Radford et al. 1998).

### Control stainings

DAPI—whole seeds or their compartments were stained according to Street et al. (2015) with some modifications: 0.1% DAPI (Sigma, D9542) in PBS buffer for 5–15 min, washed three times in PBS buffer, and then mounted on microscope slides in the same buffer. After washing, the material was observed in the epifluorescence microscope. The fluorochrome was excited by a UV-2A filter (excitation wavelength 330–380 nm). Under such conditions, the emission of DAPI was collected at 420 nm, producing a blue fluorescence (Fig. 1b).

Calcofluor white—seeds and embryos were incubated with 0.01% calcofluor white in distilled water (Sigma, F3543) for 10–15 min in the dark (according to Dumont et al. 2016). After washing with distilled water, the material was observed in CLSM (excitation wavelength 405 nm, emission wavelength 425–475 nm). Optical sections of embryos were collected and merged into one picture (Fig. 1c).

### Control experiments

In control experiments, used fluorochromes were tested not only on *S. acre* but also on other plants, and results are presented in Supplementary material. Studies on CMNB-F distribution after uncaging were performed on onion epidermal cells and *S. acre* seed (Suppl. Fig. S1). Analysis of the distribution of HPTS was performed on the example of *Arabidopsis thaliana* zygotic embryos in different stages of development (Suppl. Fig. S2). FRAP experiments were performed on the stem and leaf epidermis of *S. acre* (Suppl. Fig. S3).

### Data analysis

The images obtained with CLSM were prepared for publication with the use of ImageJ software (at least five optical sections were merged to one *z*-stack projection). The images from the epifluorescence microscope were prepared for publication with the use of Corel PHOTO-PAINT software (brightness and contrast were adjusted).

### The criteria for determining the embryo developmental stages

The criteria for determining the developmental stages of *S. acre* seeds are summarised in Table 2. The main criterion was not only the shape of the embryo but also the whole embryo dimension and the cotyledon length. Mature embryos were not analysed due to the possibility of entry into dormant phase.

**Table 2:**
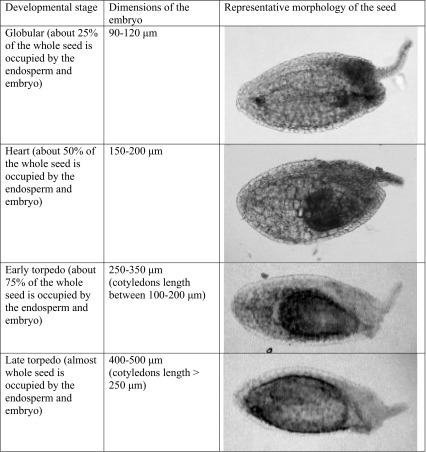
The criteria for determining the developmental stages of *Sedum acre* seeds

### Fluorochrome movement frequencies

The frequencies of fluorochrome movement between different seed compartments were scored and assessed according to the method described by Kim et al. (2002). Briefly, the number of seed compartments (which allowed movement of symplasmic transport fluorochromes) was calculated and compared to the whole seed compartments tested. Afterwards, the percentage of such ratios was calculated (Table 3). For simplification, the haustorial suspensor consisting of the giant basal cell with a micropylar haustorium and a few smaller chalazal cells is called a basal cell in the table.

**Table 3:** Fluorochrome movement between seed compartments analysed during the studies

Seed compartments	Developmental stages			
	Globular, %	Heart, %	Early torpedo, %	Late torpedo, %
Embryo proper	87.5 (7^a^/8^b^)	90 (9^a^/10^b^)	92.9 (13^a^/14^b^)	86.7 (13^a^/15^b^)
Basal cell/embryo proper	77.8 (7^a^/9^b^)	66.7 (2^a^/3^b^)	50 (1^a^/2^b^)	Not analysed
Embryo proper/basal cell	9.1 (1^a^/11^b^)	0 (0^+^/13^b^)	10 (1^+^/10^b^)	Not analysed
Basal cell/endosperm	77.8 (7^a^/9^b^)	88.9 (8^a^/9^b^)	100 (3^a^/3^b^)	Not analysed
Endosperm/basal cell	16.7 (3^a^/18^b^)	17.6 (3^a^/17^b^)	33.3 (3^a^/9^b^)	Not analysed
Funiculus/external integument	97.3 (36^a^/37^b^)	100 (11^a^/11^b^)	100 (8^a^/8^b^)	100% (27^a^/27^b^)

“Not analysed” means that at that stage of seed development, the basal cell starts to degenerate or is already dead (Kozieradzka-Kiszkurno and Bohdanowicz 2006)

“Can be assessed only indirectly” means that the chalazal suspensor cells dimensions do not allow for introducing the fluorochrome directly into them

“Basal cell/embryo proper” and “embryo proper/basal cell” should be understood to mean that between the basal cell and embryo proper are chalazal suspensor cells

^a^Number of seeds/embryos allowing movement of symplasmic transport fluorochromes between the mentioned compartments

^b^Number of seeds/embryos tested

## Results

### Fluorochrome movement between embryo cells in different developmental stages

**Fig. 2:**
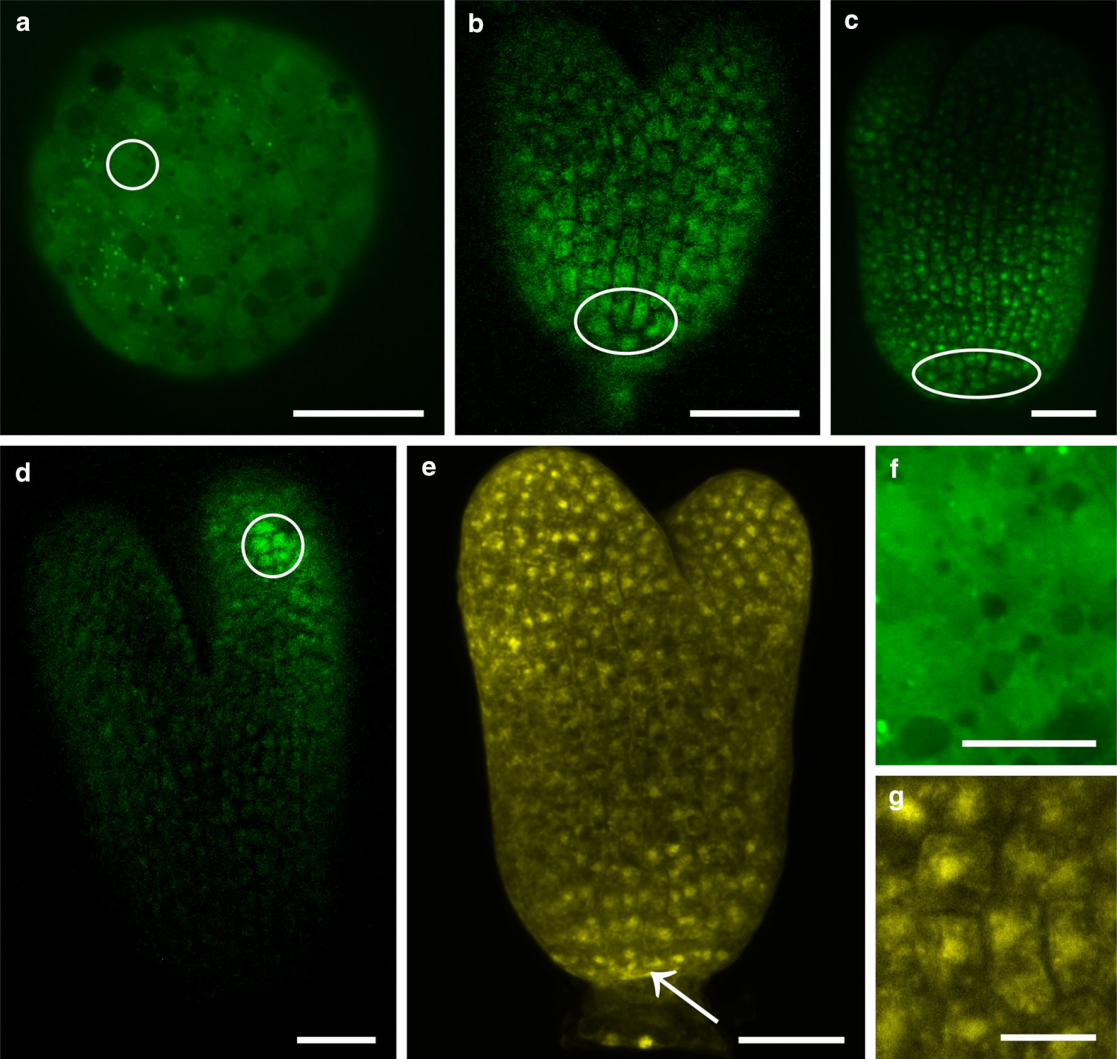
Symplasmic communication in *Sedum acre* embryos after CMNB-caged fluorescein and LYCH treatment. Both fluorochromes were present in all embryo cells at different developmental stages: globular (**a**), heart (**b**), early torpedo (**c**) and torpedo (**d**, **e**). Fluorescein (**a**–**d**, **f**) moved from the place of uncaging (*white circles* and *ellipsoids*) to other embryo cells. LYCH (**e**, **g**) was applied to a few cells at the root pole (*arrow*) and was observed in other cells of the embryo. Both fluorochromes were present in the cytoplasm (**f**), but nuclear localization also occurred (**g**). All images are CLSM *z*-stack projections consisting of at least five optical sections. *Scale bar* 30 μm (**a**), 50 μm (**b**–**e**), 15 μm (**f**–**g**)

Independently of the embryo developmental stage (see Table 2) and the embryo area where the CMNB-F uncaging was performed, distribution of fluorescein (332 Da) was uniform in all embryo cells (Fig. 2a–d). Such a fluorochrome distribution indicates that an embryo is a single symplasmic domain at each developmental stage, because fluorescein moved from the place of release to other embryo cells. The same pattern of distribution was gained in the case of LYCH (443 Da; Fig. 2e) and HPTS (520 Da; data not shown) treatments, which means that PD size exclusion limit (SEL) of embryo cells is at least as high as about 0.5 kDa. Fluorochromes were located in the cytoplasm, but also nuclear localisation was noticed (Fig. 2f, g, respectively).

### Symplasmic communication between seed compartments

As ultrastructural analysis of the plasmodesmata occurring between the basal cell and chalazal suspensor cells and between the basal cell and the endosperm showed that PD were occluded by the electron-dense material located on the basal cell side and their functionality was never checked, the first question was: do fluorochromes move between these compartments?

#### Basal cell/chalazal suspensor cells/embryo proper

**Fig. 3:**
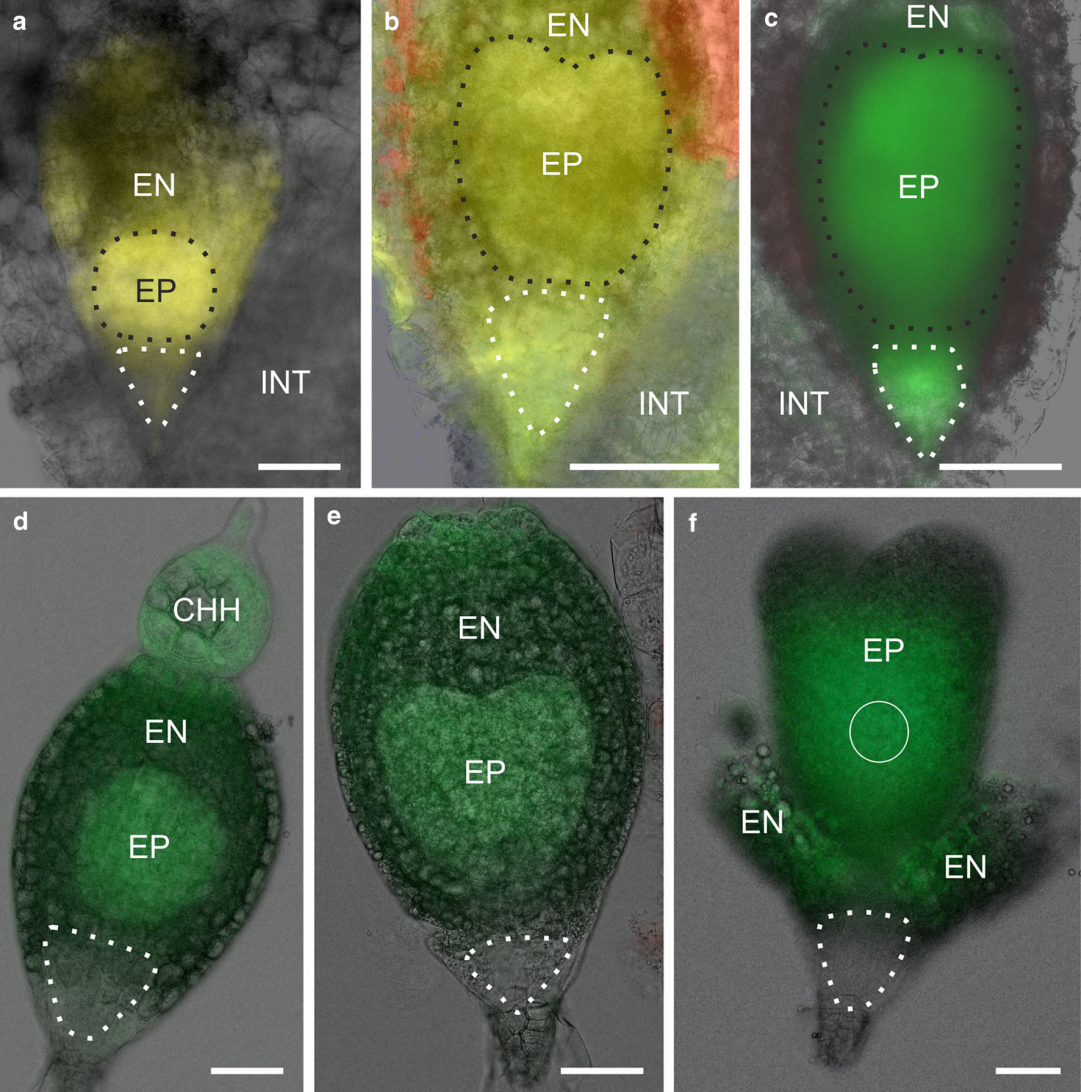
Symplasmic communication between the basal cell and embryo proper. Symplasmic communication between the suspensor and embryo proper was examined at three developmental stages: globular (**a**, **d**), heart (**b**, **e**) and early torpedo (**c**, **f**). When LYCH (**a**, **b**) and HPTS (**c**) were introduced to the basal cell, they were present in the embryo at all developmental stages. Surprisingly, symplasmic communication was restricted in the opposite direction. When the endosperm enclosing the embryo was incubated in HPTSA, the fluorochrome was present in the endosperm and embryo, but not in the basal cell (**d**, **e**). Moreover, after CMNB-F uncaging in embryo cells (*white circle*), fluorescein was visible in embryo cells and some endosperm cells, but not in the basal cell (**f**). *CHH* chalazal haustorium, *EN* endosperm, *EP* embryo proper, *INT* integument (external and internal). *White*, *dotted line* indicates the shape of the basal cell and *black*, *dotted line* indicates the shape of the embryo proper. Each image is a combination of two pictures: an epifluorescence image and a bright-field image. First, the bright-field image was changed into a *gray scale* image. After that, its transparency was set up to 50% or more, and then, it was placed upon the epifluorescence image. *Scale bar* 50 μm (**a**, **d**–**f**), 100 μm (**b**, **c**)

Fluorochromes (LYCH, HPTS) introduced into the basal cell enter the embryo proper independently of the developmental stages studied. Fluorochrome movement from the basal cell (BC) to the embryo proper (EP) was detected during the globular, heart and early torpedo stage of development (Fig. 3a–c; Table 3), which means that PD are functional in the direction BC–EP. This also means that symplasmic communication takes place between the BC and the chalazal suspensor cells (CHS), as the fluorochrome was detected in the EP. When fluorochrome was applied to the EP, it was not detected in the BC independently of the developmental stage (Fig. 3d–f; Table 3). Such a result indicates that the fluorochromes movement through PD from the EP to the BC is limited.

Thus, symplasmic tracers movement occurs longitudinally from the basal cell to the embryo proper, but in the opposite direction, it is restricted or not detectable on the light-microscopy level.

#### Basal cell/endosperm

**Fig. 4:**
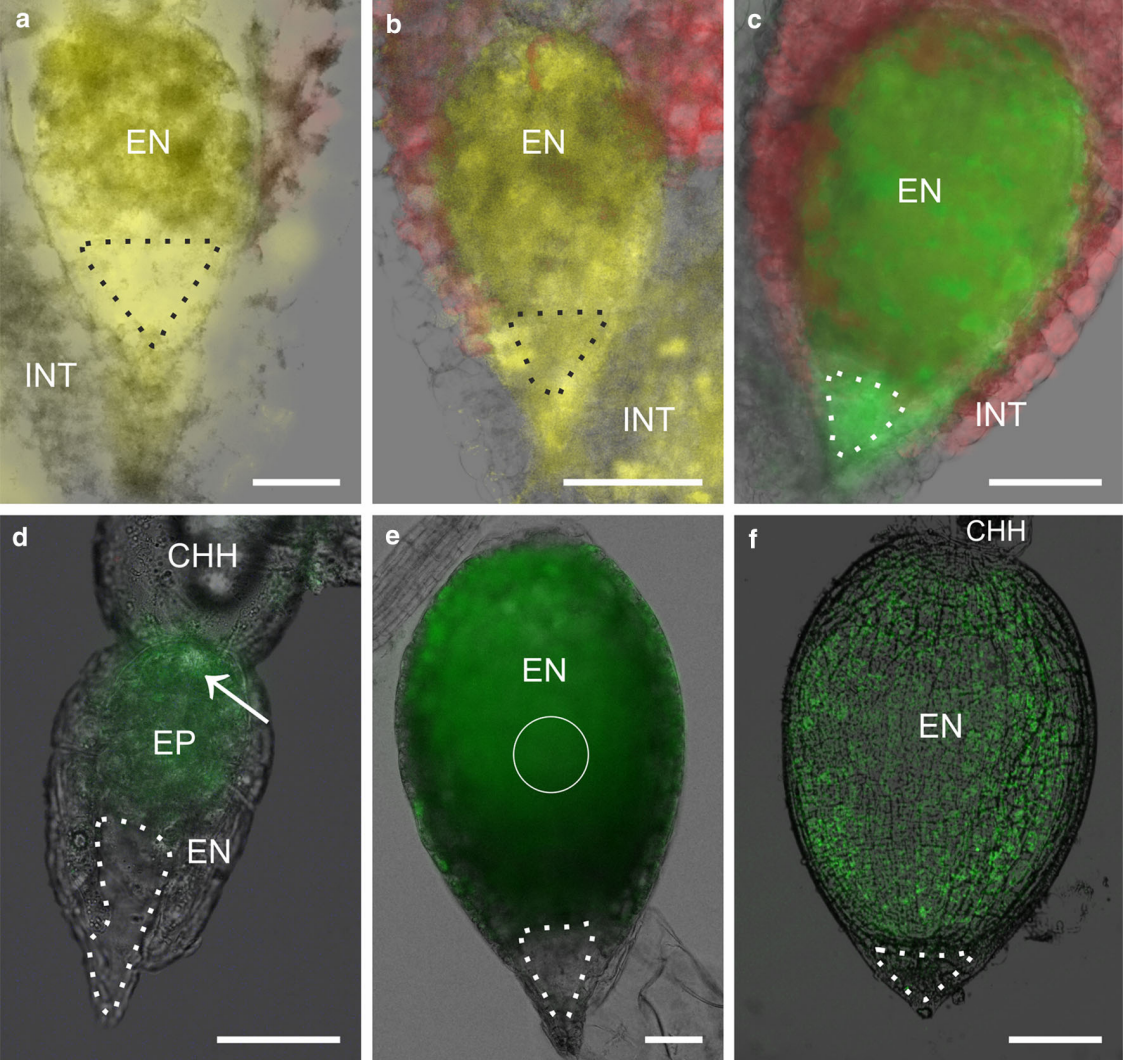
Symplasmic communication between the basal cell and endosperm. Three developmental stages were tested: globular (**a**, **d**), heart (**b**, **e**) and early torpedo (**c**, **f**). Introducing LYCH (**a**, **b**) or HPTS (**c**) into the basal cell resulted in the presence of the fluorochrome in endosperm cells independently of developmental stage (**a**–**c**). However, fluorochrome movement in the opposite direction was restricted. Independently of the fluorochrome used and technique of application: HPTS introduced by a microcapillary (*arrow*, **d**), uncaging fluorescein in endosperm cells (*white circle*, **e**) or incubation of the endosperm with embryo in HPTSA (**f**), results were repetitive. *CHH* chalazal haustorium, *EN* endosperm, *EP* embryo proper, *INT* integument (external and internal). *White, dotted line* indicates the shape of basal cell. Images “**a**”–“**e**” were taken under an epifluorescence microscope. Image “**f**” is a single CLSM optical section. All images are combinations of two pictures: a fluorescence image and a bright-field image. Merging method was described earlier (see Fig. 3). *Scale bar* 50 μm (**a**, **d**–**e**), 100 μm (**b**, **c**, **f**)

The presence of PD on the BC/endosperm border made it necessary to check what the symplasmic communication between these two compartments looks like. Fluorochromes applied to the basal cell were detected in the endosperm cells as long as the BC was not degenerated (Fig. 4a–c; Table 3). If fluorochromes were introduced into the endosperm, fluorescence was not detected in the basal cells independently of the developmental stage (Fig. 4d–f; Table 3).

Thus, the symplasmic communication on the “radial” direction from the basal cell to the endosperm takes place, but in the opposite direction, it is restricted or not detectable on the light-microscopy level.

#### Funiculus/seed coat

**Fig. 5:**
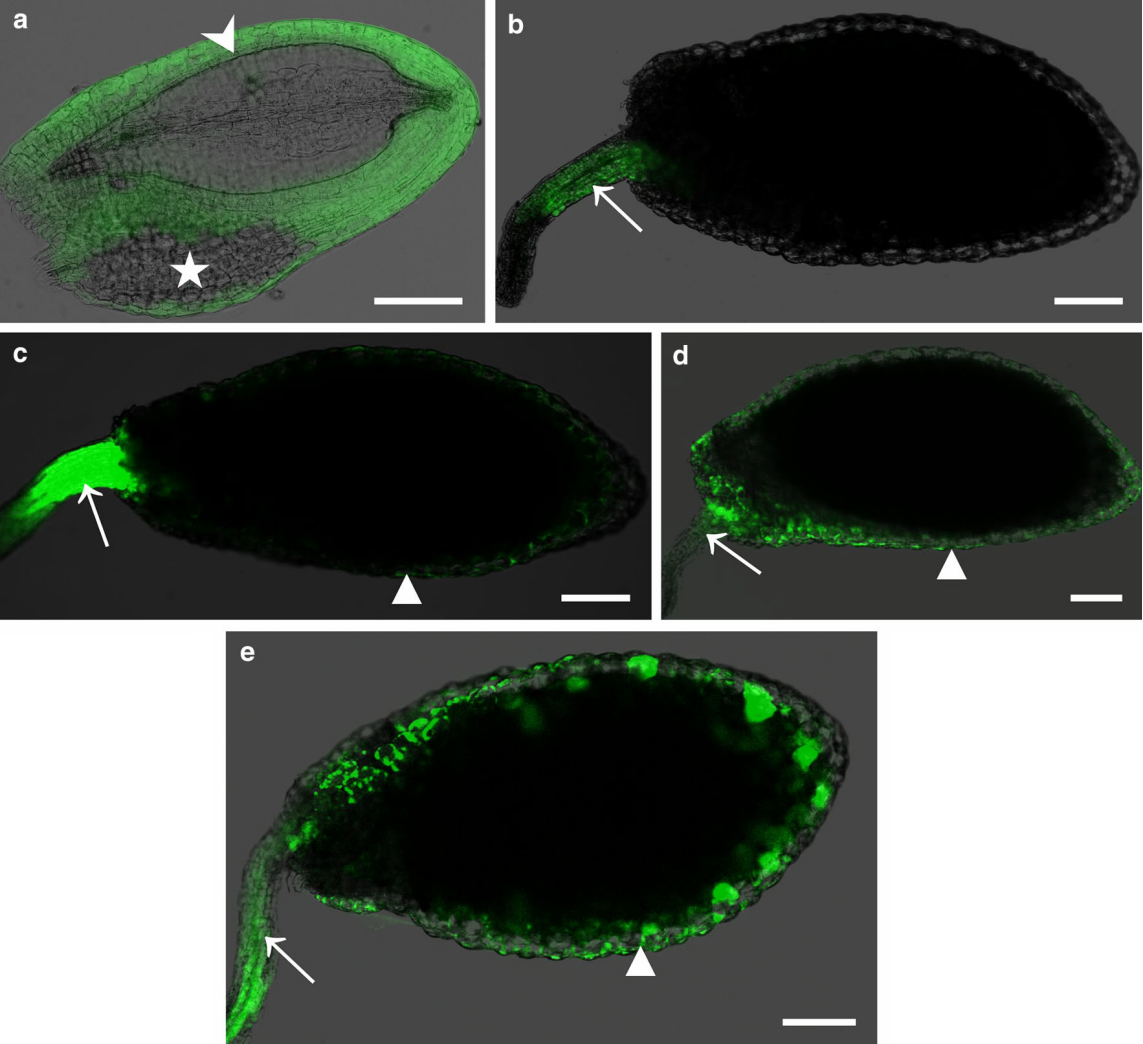
Symplasmic transport of HPTS within the seed and between a mother plant and seeds. When HPTS was introduced into a few cells of external and internal integument (*arrowhead,* **a**), it was present within the seed-coat cells except the micropylar pocket (*asterisk*, **a**). After incubation of inflorescence stems in HPTSA, its esterase-cleaved form (HPTS) was visible only in the funiculus (**b**) or in the funiculus and external integument (**c**–**e**). Location of the fluorochrome depended on the time of incubation. When stems were immersed for 80 min, fluorochrome was observed only in the funiculus (**b**). Longer times of incubation: 3 h (**c**), 6 h (**d**), 48 h (**e**) resulted in the presence of fluorochrome not only in the funiculus but also in the external integument. A *white arrow* indicates the funiculus and *white triangle* marks the external integument. Image “**a**” was taken under an epifluorescence microscope. Images “**b**”–“**e**” are CLSM *z*-stack projections reconstructed from at least five optical sections. *Scale bar* 100 μm

Two sets of experiments were performed to check symplasmic communication between the seed coat and other seed compartments. In the first one, HPTS was introduced into a few external and internal integument cells (by a microcapillary). This resulted in the presence of fluorochrome within all external and internal integument cells except the area called the “micropylar pocket’’ (a term used by Roberts et al. 2003; Fig. 5a). In the second set of experiments, HPTS was introduced into the funiculus (by a phloem transport from the stem which was immersed in HPTSA solution) and the presence of fluorochrome was detected in the close proximity to the end of the vascular bundle of the funiculus and in the external integument. The internal integument, the endosperm and the embryo together with the suspensor were not filled with the fluorochrome (or the amount of the fluorochrome was too low to detect it in CLSM and epifluorescence microscope) applied to the funiculus independently of treatment time (Fig. 5b–e; Table 3). These results suggested that there was symplasmic communication between the mother plant and seeds, but it was restricted to the external integument.

## Discussion

The above results suggest that symplasmic communication between *Sedum* seed compartments and the embryo proper is not uniform. This is the first time such a detailed description of the low-molecular-weight symplasmic tracers distribution during the development of *S. acre* seed has been presented.

### Embryo proper

Symplasmic communication during the zygotic embryogenesis was performed mainly on *A. thaliana*. The first paper on this subject proved that the embryo from the globular to torpedo stage was a single symplasmic domain for low-molecular-weight fluorochromes (Kim et al. 2002). In the case of *S. acre,* the embryonic pattern of symplasmic communication is similar to that described for *Arabidopsis*. At any developmental stage, low-molecular-weight fluorochromes freely moved between embryo cells which means that the *S. acre* embryo is a single symplasmic domain. Thus, our results are another example showing that the symplasmic communication during zygotic embryogenesis is uniform with respect to low-molecular-weight substances. Moreover, symplasmic communication during *S. acre* embryogenesis is the same as in the classical *Capsella* variation of the Onagrad type and Caryophyllad type of embryonic development, which could suggest that such pattern of symplasmic communication is universal. To check if SEL of plasmodesmata also decreases, as in case of *Arabidopsis*, in *S. acre* during embryo development, studies which use fluorescent-labelled dextrans with higher molecular weight should be performed in the future.

### Embryo symplasmic domains

In *S. acre*, the embryo consists of a haustorial suspensor built by a giant basal cell with a micropylar haustorium and a few smaller chalazal cells and the embryo proper (Kozieradzka-Kiszkurno and Bohdanowicz 2003, 2006; Kozieradzka-Kiszkurno et al. 2011a, b; Kozieradzka-Kiszkurno and Płachno 2013). Ultrastructural analysis of the embryo has shown the presence of unusual PD between the basal cell and chalazal suspensor cells and between the basal cell and the endosperm (Kozieradzka-Kiszkurno and Bohdanowicz 2010; Kozieradzka-Kiszkurno et al. 2011a; Kozieradzka-Kiszkurno and Płachno 2012). The results presented here showed that symplasmic tracers moved from the basal cell to the embryo proper and from the basal cell to the endosperm, but fluorochromes were not detected in the basal cell when were applied to the embryo or endosperm. What can such findings suggest? In the first place, it raises the supposition of one-way communication between these seed compartments.

Unidirectional movement through plasmodesmata has been described in the case of the leaf trichomes of tobacco (Christensen et al. 2009). Fluorescent probes introduced into the basal trichome cell were not able to enter the epidermis, but fluorescent probe movement in the opposite direction took place. Authors point out that such results provide evidence for unidirectional transport through PD at a specific cell–cell interface which, in this case, was the epidermis/trichome boundary (Christensen et al. 2009). The results presented here may be considered as another example of unidirectional movement through PD, but with great caution. Namely, it must be taken into consideration that the movement of tracers molecules can be used as an indicator of symplasmic continuity between different seed compartments/cells, but absence of fluorochromes movement cannot be considered as a sufficient argument for a one-way transport through plasmodesmata. There could be many other reasons for unidirectional movement independent of plasmodesmata functionality. As the movement through PD takes place by diffusion, all the factors which influence this process may influence the tracers movement between analysed *S. acre* seed compartments. The movement in the symplasm is caused by concentration differences in combination with electric potential gradients and by gravity (Arisz 1969). The PD function depends on external and internal factors such as even metabolic status (Tucker 1993; Schulz 1995; Wright and Oparka 1997) or cytosolic Ca^+2^ levels (Tucker 1988; Tucker and Boss 1996; Holdaway-Clarke et al. 2000), and many others (Christensen et al. 2009). Consideration of presented results as an example of a one-way movement through PD is very tempting, but it requires further experiments in the future. Primarily, some experiments with metabolic inhibitors should be carried out, as it was shown that they alter the mode of solute flow through PD (Christensen et al. 2009).

In *S. acre*, interestingly, the restriction of fluorochrome movement, if takes place, proceeded in the direction of the basal cell which possesses occluded PD. It can be speculated that this occlusion, the chemical character of which is not determined yet, is a structural basis for the restriction in symplasmic communication on the basal cell/endosperm border. The same factor may concern the symplasmic communication along the longitudinal embryo axis. Namely, the most occluded PD are between the basal cell and the first layer of chalazal suspensor cells (Kozieradzka-Kiszkurno and Bohdanowicz 2010; Kozieradzka-Kiszkurno et al. 2011a). For the complete explanation, how symplasmic coupling of these seed compartments operates the chemical nature of occlusion must be determined.

Restriction in the symplasmic communication between the suspensor and the embryo proper during development has been described for *A. thaliana* (Stadler et al. 2005). It was shown that symplasmic communication between the suspensor and embryo proper occurs at least in one direction (from the suspensor to the embryo proper), but such communication is temporal and depends on the stage of embryo development. Stadler et al. (2005) showed that symplasmic connectivity between the embryo and suspensor diminished and was even disrupted during the hypophysis specification (Stadler et al. 2005), which is much earlier in comparison to *S. acre*, where the movement from the basal cell takes place up to the early torpedo stage. The differences between the results obtained for *A. thaliana* and *S. acre* may be a consequence of the different morphologies and vitality of the suspensor in both species.

Another explanation for the detected differences in the pattern of the symplasmic movement between *A. thaliana* and *S. acre* may rely on the ultrastructure of PD. In *S. acre*, PD in the suspensor are not only occluded, but also branched (Kozieradzka-Kiszkurno and Bohdanowicz 2010). In *Arabidopsis*, PD are simple (Kim et al. 2002). It is postulated that branched PD occur in the mature cells where they regulate more precisely the movement of molecules between cells (Burch-Smith and Zambryski 2012). The earlier question about the involvement of branched PD in directionality in symplasmic transport (Zambryski and Crawford 2000) is still unanswered, but results presented here may offer an answer.

### Seed symplasmic domains

From the viewpoint of phloem transport, the seed is the most important sink part within a plant body, and that is why phloem transport of nutrients to the seed has been investigated extensively. However, data concerning symplasmic communication between seed compartments are scarce.

The analysis of HPTS movement has shown that in *Pisum sativum* (L.), solutes imported by the phloem move to the chlorenchyma and ground parenchyma but not to the branched parenchyma as a result of post-phloem symplasmic transport of nutrients which demonstrates that HPTS moves from the sieve elements of the chalazal vein symplasmically (van Dongen et al. 2003). Patrick et al. (1995) showed that there was symplasmic communication only between sieve elements of the vascular web and the ground parenchyma in *P. vulgaris* seeds. In *V. faba,* the fluorochrome movement took place from sieve elements into the chlorenchyma and the innermost layer of the parenchyma. Despite the differences in fluorochrome distribution in the above types of seeds, it is clear that the chalazal end and seed coat compartments were connected by PD. Thus, these results show that delivering the nutrients to the filial tissues (the embryo and endosperm) is performed by symplasmic transport (Patrick and Offler 2001; Stadler et al. 2005). Studies on *A. thaliana* have revealed that in seeds, the phloem-unloading domain is located at the end of the funicular phloem and that the entire outer integument is a symplasmic extension of the phloem and that the inner integument and globular embryo with the suspensor are symplasmic domains (Stadler et al. 2005). These results have been interpreted to mean that there are neither functional PD on this border nor are the two integuments connected by a significantly large number of PD (Stadler et al. 2005). Our results showed that in *S. acre* seeds, HPTS was unloaded from the phloem into the external integument cells and not further. Thus, similar to *A. thaliana*, the external integument is an extension of the phloem, whereas the internal integument, endosperm and embryo are domains symplasmically isolated from the maternal phloem. However, it must be taken into consideration that not detecting the fluorochrome is not a sufficient argument for symplasmic isolation (see “Embryo symplasmic domains”). Additional studies have to be carried out to confirm limited fluorochrome movement between the maternal phloem and *S. acre* seed compartments.

In conclusion

**Fig. 6:**
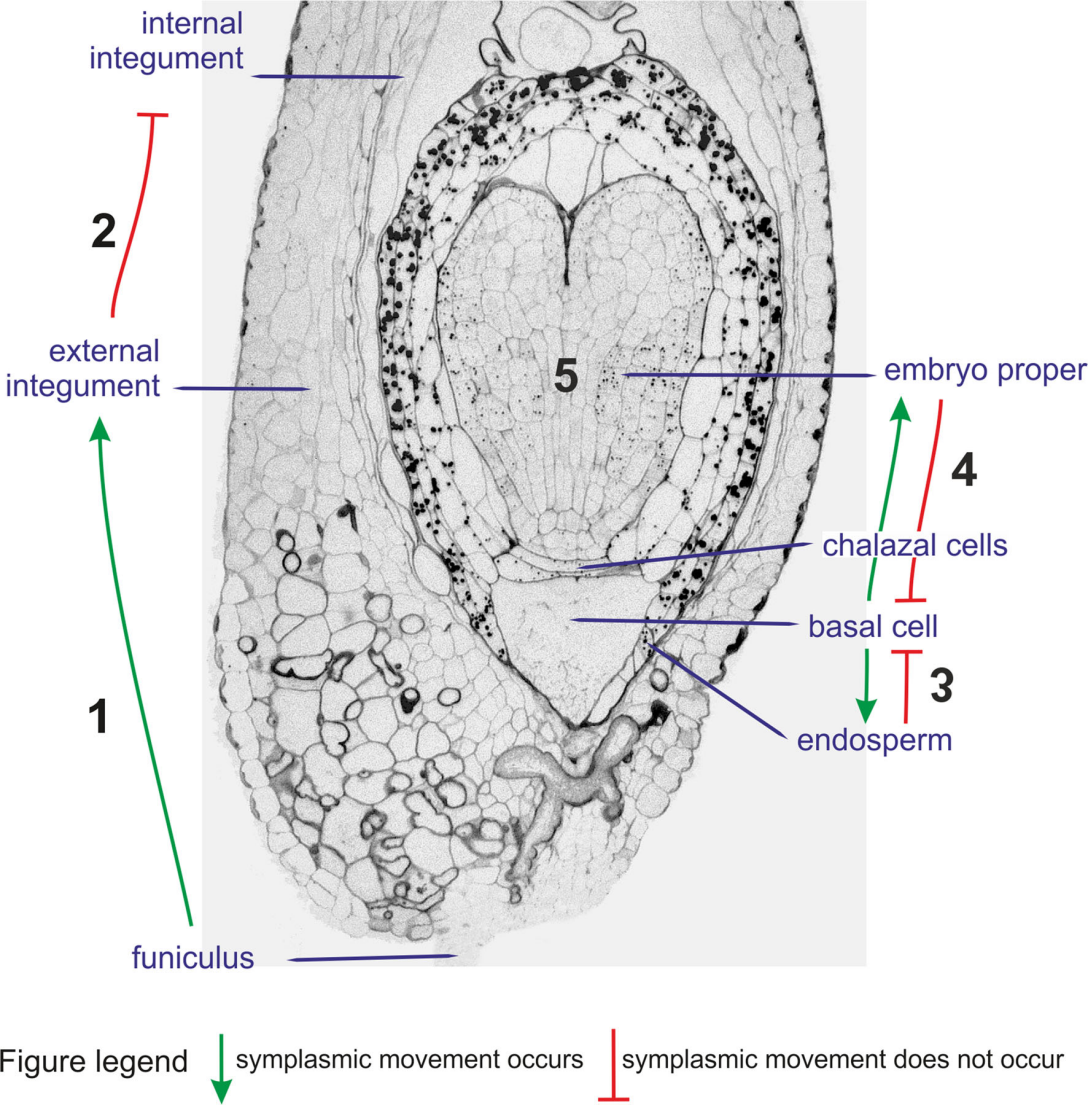
Functionality of plasmodesmata during *Sedum acre* seed development—scheme. *Arrows* show the direction of symplasmic movement between seed compartments and embryo compartments. *Numbers* mark symplasmic domains identified within *Sedum acre* seed

The results show that (Fig. 6):

1. there is symplasmic communication between the funiculus and external integument,
2. the external and internal integument are symplasmically isolated,
3. differences in the tracers movement from the basal cell to the embryo and in the opposite direction, may indicate (though it is not irrefutably proven) that both directions are not equivalent with respect to symplasmic communication,
4. the embryo is a single symplasmic domain independent of the developmental stage,
5. identified symplasmic domains refer to molecules whose molecular weight is about 0.5 kDa.

## Future perspectives

Concerning the results described in the present paper, it appears that further ultrastructural studies of PD are needed. It is important to know how PD are distributed on borders between seed compartments. The structure of these PD should also be examined using an antibody against callose, because this compound can be a key regulator of their functionality. Moreover, experiments with molecules of higher molecular weight must be performed to determine the maximal SEL of PD in different developmental stages of *S. acre* seed.

### *Author contribution statement*

All authors contributed to the conception and design of the manuscript. EK and J W-M conducted experiments, analysed data and wrote the manuscript. All authors discussed the results and commented, corrected and approved the manuscript.

## Electronic supplementary material

Below is the link to the electronic supplementary material.

Supplementary material 1 (TIFF 30107 kb)

Supplementary material 2 (TIFF 25261 kb)

Supplementary material 3 (TIFF 16797 kb)
