# Supplementary figures and images for: Identification of symplasmic domains in the embryo and seed of Sedum acre L. (Crassulaceae)
Source: Planta. 2016 Nov 25;245(3):491–505. doi: 10.1007/s00425-016-2619-y (PMC5310571; doi:10.1007/s00425-016-2619-y)

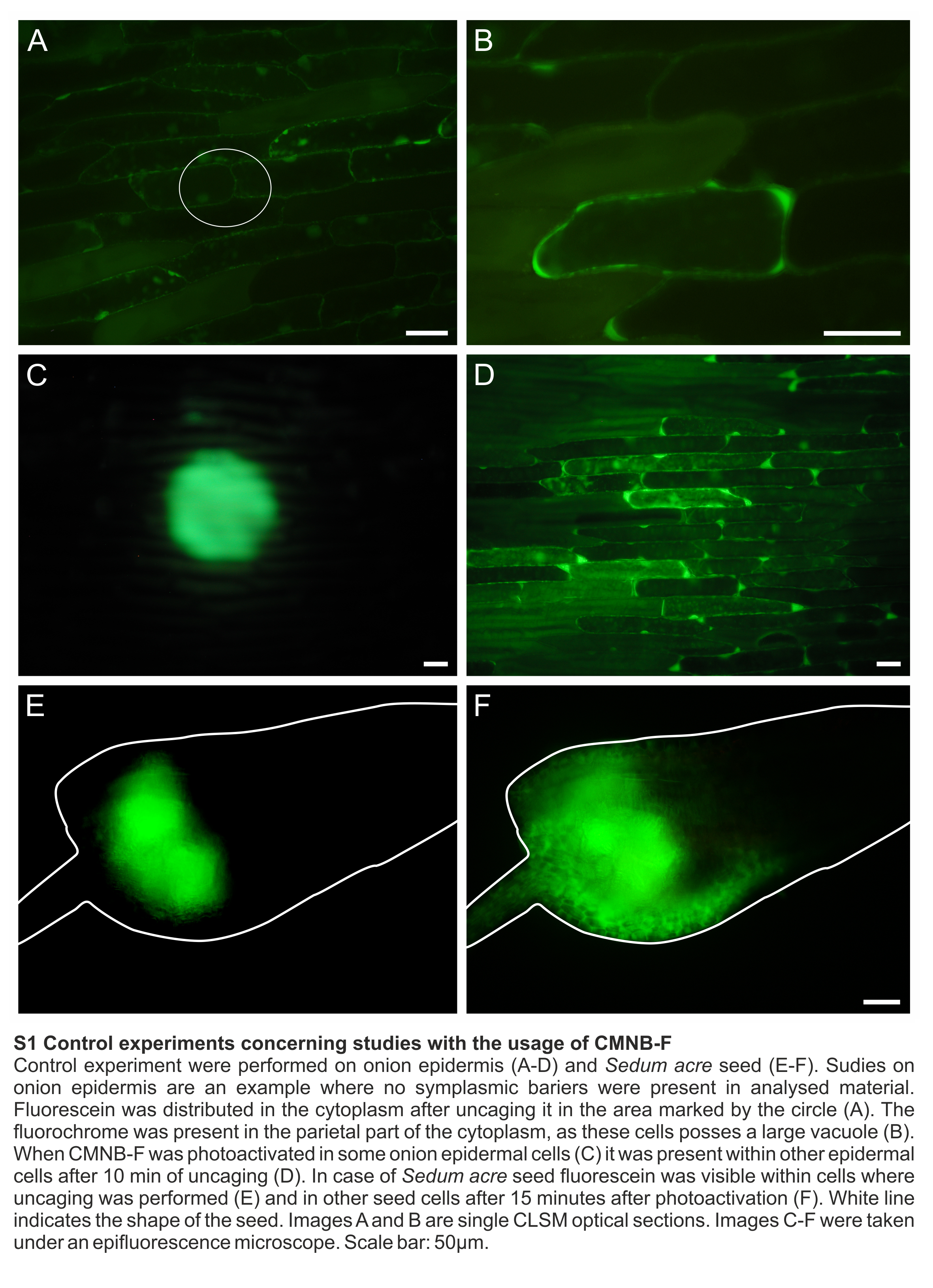

Supplement: Supplementary file 1 — Supplementary material 1 (TIFF 30107 kb) [file 425_2016_2619_MOESM1_ESM.tif]

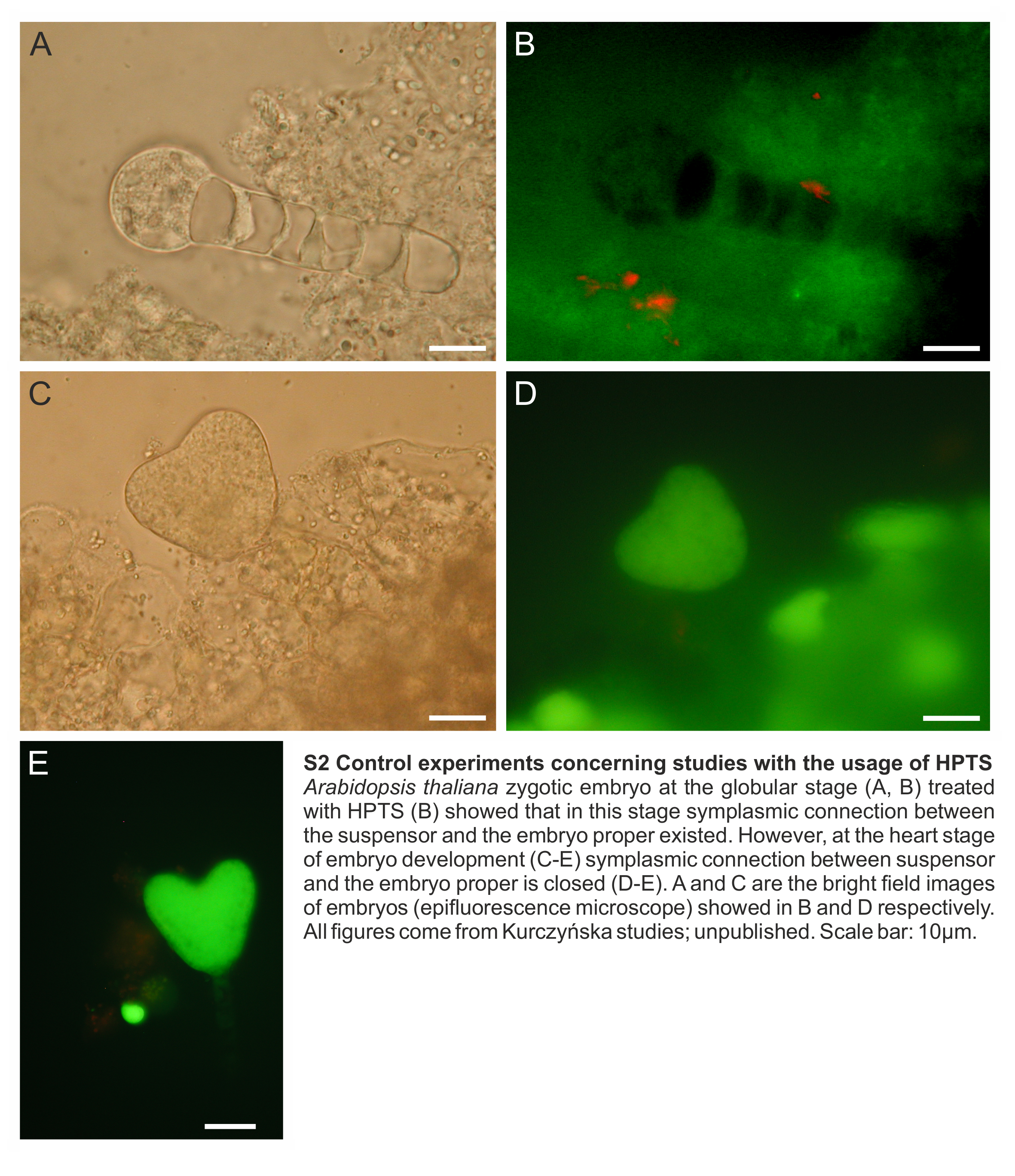

Supplement: Supplementary file 2 — Supplementary material 2 (TIFF 25261 kb) [file 425_2016_2619_MOESM2_ESM.tif]

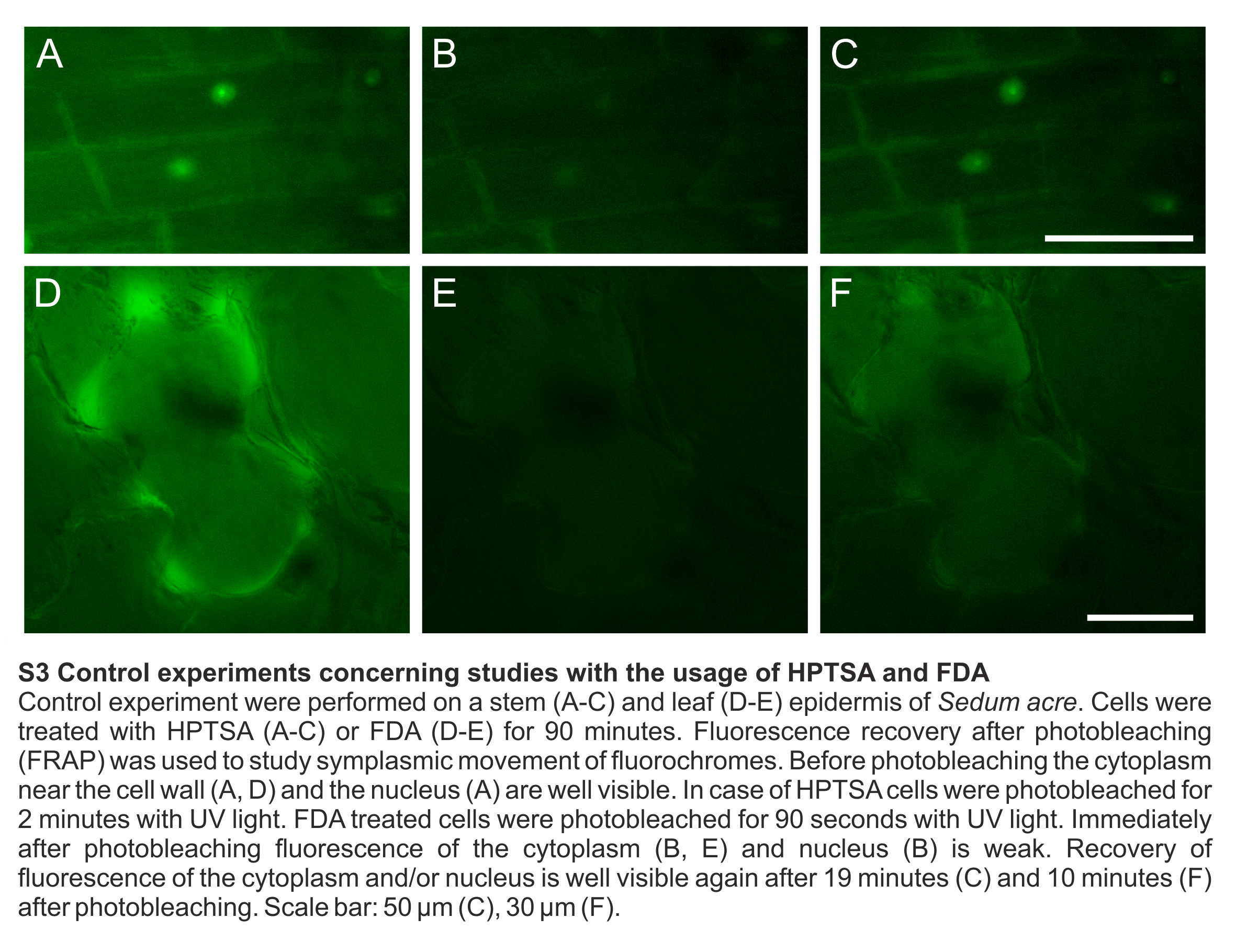

Supplement: Supplementary file 3 — Supplementary material 3 (TIFF 16797 kb) [file 425_2016_2619_MOESM3_ESM.tif]
